# 

**Published:** 1915-07-31

**Authors:** 


Mr. Archibald Anderson, of Hyde Park, W., and Bournemouth, Hants, seventy-nine, Registrar of Friendly Societies for Scotland, 1874-9, has left £1,000 each to the Hospital for Paralysis and Epilepsy, Queen Square, W.C., and the London Homœopathic Hospital, and smaller sums to four other charities.

### Relieving Pain on the Battlefield.

" The question of relieving the pain of the wounded is considered very briefly in our ' Training,' and it states that an opiate should only be given under the orders of a medical officer. Personally I see no reason why a well-trained regimental bearer before going into action should not be supplied with a small tube containing one-third grain tabloids of morphia, with instructions to give one to each severely wounded man he attends to. . . . Such treatment, if intelligently carried out, abolishes much of the physical and mental agony which our soldiers undergo in default of this relief. . . Which would any of you officers prefer—hours of pain, or an injection of morphia followed by several hours' release from mental anxiety and physical pain but attended by the remote possibility of an abscess forming at the point of injection? I know which of the two I would choose."—*Major Ensor, in Lecture before United Services Medical Society*, 1913.

### Buildings Contemplated and in Progress.

Barr Beacon.—The West Bromwich Town Council have agreed to join with the Walsall Corporation in purchasing a site of about thirty-nine acres near Barr Beacon, at £30 per acre, for building a tuberculosis sanatorium and hospital.

Highbridge.—The Urban District Council have agreed to attend a joint conference of the Bridgwater Rural District Council and the Burnham Urban District Council to consider the provision of a joint hospital for the district.

Huddersfield.—A military hospital will be built at Huddersfield for 500 wounded soldiers. The cost, about £15,000, will be defrayed by voluntary subscriptions. The Town Council will give a site on their estate at Royds Wood, and will also provide the hospital with electricity, gas, sewers, and water free of charge.

Langwith.—The Derbyshire County Council have decided to ask the Local Government Board to hold an inquiry into an application of the North Derbyshire Joint Hospital Committee for a loan of £16,750 for extending the Morton, Dronfield, Maston Moor, and Langwith Isolation Hospitals.

Penmore.—The Derbyshire County Council have resolved to apply to the Local Government Board for sanction to a loan of £6,000 for the extension of the administrative block and a new ward at Penmore Hospital.

Swansea.—The Health Committee of the Corporation have decided to provide a new isolation hospital and to apply for sanction to a loan. About £40,000 is involved.

### Gifts and Bequests.

Miss Elizabeth Browne, of Clanricarde Gardens, Tunbridge Wells, has left £1,000 to the Protestant Orphan Institution, Dublin; £500 to the General Hospital, Tunbridge Wells; and £100 to the London Hospital.

Mrs. Isabella Gibb or Ure, of Cairndhu, Helensburgh, N.B., mother of Lord Strathclyde (formerly known as Mr. Alexander Ure, K.C.), left £100 to the Victoria Infirmary, Helensburgh, and £50 to the Children's Home, Helensburgh.

Lieutenant-Colonel Arthur Percival Dearman Birchall, Royal Fusiliers, commanding 4th Battalion, Canadian Infantry, who was killed in action on April 23, aged thirty-eight, has bequeathed £200 each to the London Hospital and the National Institution for the Blind, and £100 each to the Gloucester Royal Infirmary and the Children's Hospital, Halifax, Nova Scotia.

Rates of Subscription: Twelve months, 6/6; Six months, 4/-; Three months, 2/-, post free to any address in the United Kingdom. Abroad, Twelve months, 8/8; Six months, 5/-; Three months, 2/6, post free.—Address The Subscription Dept., "The Hospital," The Hospital Building, 28/29 Southampton Street, Strand, London, W.C.

